# Peri-Implantitis

**DOI:** 10.3390/dj12080251

**Published:** 2024-08-09

**Authors:** Miriam Ting, Jon B. Suzuki

**Affiliations:** 1 Department of Periodontics, School of Dental Medicine, University of Pennsylvania, Philadelphia, PA 19104, USA; 2 Think Dental Learning Institute, Paoli, PA 19301, USA; 3 Department of Graduate Periodontics, University of Maryland, Baltimore, MD 20742, USA; jsuzuki@temple.edu; 4 Department of Graduate Prosthodontics, University of Washington, Seattle, WA 98195, USA; 5 Department of Graduate Periodontics, Nova Southeastern University, Fort Lauderdale, FL 33314, USA; 6 Department of Microbiology and Immunology (Medicine), Temple University, Philadelphia, PA 19140, USA; 7 Department of Periodontology and Oral Implantology (Dentistry), Temple University, Philadelphia, PA 19140, USA

**Keywords:** dental implants, peri-implantitis, peri-implant mucositis, implant failures

## Abstract

Peri-implantitis can affect the longevity of successfully integrated implants. Implant success is dependent on reducing the peri-implantitis risk or successfully managing peri-implantitis. Further understanding of peri-implantitis can be derived from its prevalence, microbial and diagnostic findings, existing therapies, and the effects of systemic health issues and medication. Based on published information: (1) peri-implantitis is higher in patients who have periodontitis or smoke as well as in implants with 5 years of function; (2) peri-implantitis microflora is different from periodontitis; (3) peri-implantitis risk is increased in patients with cardiovascular diseases and uncontrolled diabetes; (4) most reported peri-implantitis therapies may result in resolution, but the best peri-implantitis treatment is still to be determined; (5) more frequent peri-implant maintenance may reduce risk for peri-implantitis.

## 1. Introduction

Dental-implant-supported fixed restorations of full and partial edentulism are predictable alternatives to removable dentures and fixed tooth-supported bridges [1,2]. Dental implants reported 92.8–97.1% survival rates but can be prone to peri-implantitis during a patient’s lifetime [3,4]. Peri-implantitis may damage the soft and hard tissue around dental implants leading to bone loss, periodontal pocketing, and loss of osseointegration around the implant [5]. An understanding of the available research on peri-implantitis can improve clinical care and reduce the risk of implant failure [6,7].

## 2. Definition of Peri-Implantitis

Peri-implantitis is defined inconsistently in research studies and clinical reports. These are some common peri-implantitis definitions: (1) the classification of implant diseases by American Academy of Periodontology and the European Federation of Periodontology [8]; (2) the consensus definition by the 1st European Workshop on Periodontology [9]; (3) supporting bone loss in the presence of peri-implant mucosa inflammation and bleeding on probing with or without pus [5]; (4) progressing peri-implant bone loss greater than 2 mm in presence of purulence, bleeding on probing, and greater than 6 mm probings; (5) peri-implant suppuration, bleeding, probings ≥ 5 mm, and radiographic bone loss ≥ 2.5 mm or beyond the first three threads [10]; (6) peri-implant bleeding on probing and probings > 5 mm; and (7) peri-implant mucosa inflammation with peri-implant crestal bone loss [11].

## 3. Potential Etiologies and Risk Factors

The American Academy of Periodontology and the European Federation of Periodontology jointly published a classification of peri-implantitis [12]. The new classification included implant health, peri-implant mucositis, peri-implantitis, and hard- and soft-tissue deformities. Hard- and soft-tissue deformities of dental implants may result in peri-implantitis; the potential factors include improper implant placement, inadequate bone quality, inadequate bone quantity, and traumatic occlusion [13].

Improper implant placement can result in compromised hard-tissue and soft-tissue defects around an implant; this can develop relatively quickly to bone loss around an implant and a diagnosis of peri-implantitis.

Improper surgical implant placement is an iatrogenic risk factor for peri-implantitis. Common examples of improper implant placement include being too close to a contiguous tooth or another dental implant and not having adequate 1.5 to 2.0 mm of bone buccal and lingual to the placed implant. 

Inadequate alveolar bone quality may also be a contributing factor. Bone density is classified as D1, D2, D3, and D4 bone [14]. This classification pertains to the density of bone related to trabecular and cortical bone. If implants are placed in inadequate bone quality classified as D3 and D4 bone, the implant is at a higher risk of peri-implantitis. Other situations contributing to this lack of bone quality include osteoporosis, osteopenia, or other bone diseases [14]. 

Inadequate bone quantity can be correlated to improper implant placement. Inadequate bone quantity results from lack of proper diagnosis and poor surgical implant placement into a site where the bone is insufficient to support the stability of the dental implant. 

Poor angulation and positioning of dental implants result from improper diagnosis or clinician error. Angulation and positioning of the implant that results in a thin buccal plate between the implant and the buccal bone is highly susceptible to peri-implantitis. When there is bone loss, dehiscence or fenestration of the implant can occur. 

Poor treatment planning generally leads to poor design of the implant prosthesis. The implant prosthesis with a large occlusal table can negatively impact the support of the dental implant. The implant prosthesis with poorly designed shape and contour can negatively impact the patient’s oral hygiene. In addition, appropriate structures of the implant prosthesis must be designed so that the angulation of occlusal force is parallel to the long axis of the tooth. Extensions of the implant prosthesis due to cantilevers, missing teeth, or other factors can negatively impact the occlusal factors of a dental implant. 

Occlusion is a contributing factor for peri-implantitis. Occlusal trauma has a positive correlation to increased peri-implant bone loss around dental implants [13]. Heavy implant occlusal factors like parafunction and bruxism could cause early failure of the dental implant. Occlusal overload is often regarded as one of the main causes of peri-implant bone loss and implant prosthesis failures. Radiographically, this results in crestal bone loss, mobility of the implant, and damage to the prosthesis. 

Early loading of dental implants may disrupt the physiologic osteointegration process, interfering with optimal bone remodeling. Interference with the osteointegration can result in inadequate bone formation and loss of crestal bone around a dental implant, leading to peri-implantitis.

Smoking is a well-known risk factor for multiple diseases including cancer, heart disease, and dental implant diseases. Dental implants are negatively impacted by smoking. Smoking changes the microbiome and the immune response around dental implants. Electronic cigarettes also have an adverse effect on implant success [15]. Therefore, smoking and tobacco negatively affect the outcome of virtually all therapeutic procedures, including dental implants. The failure rate of implant osteointegration is considerably higher among smokers. Oral hygiene around implants and the peri-implantitis risk are adversely affected by smoking. 

Cement negatively impacts on the overall health of a dental implant. Peri-implantitis is frequently the result of cement left around an implant prosthesis [16]. The European Federation of Periodontology consensus report and other systematic reviews concluded that cement is the most common reason for peri-implantitis [17,18].

The host response is an integral part of implant maintenance and osteointegration success [19]. Any compromised immune response may lead to improper osteointegration and inadequate host defense mechanisms against bacterial colonies around the dental implants. Different inflammatory components can also lead to excessive destructive cytokines and host response cells that can initiate peri-implantitis and ultimately impact the success of the bone-to-implant interface. 

Systemic diseases can also have a significant role in peri-implantitis and implant failures. Diabetes mellitus is the most exhaustively explored factor. The impact of diabetes and glycemic control on the osteointegration of dental implants is well recognized [20]. Successful dental implant osteointegration can be accomplished in subjects with diabetes with good metabolic control, which is a hemoglobin A1C of 7% or less. Diabetic patients with a controlled health status have a similar osteointegration pattern as subjects without diabetes mellitus. 

Osteoporosis is another systemic disease that has a major impact on implant success. Based on the DEXA dual X-ray absorptiometry and resulting T-score of the patient, we can predictably look for stability of our dental implants upon surgical placement [21]. A healthy patient with ideal bone density of a T-score within 1.5 standard deviations of the norm may improve osteointegration. In osteopenic patients with a diagnosis of 1.5 to 2.5 standard deviations from the mean, osteointegration may be successful but may take a longer time. In patients with osteoporosis with a diagnosis of 2.5 or greater standard deviations from the mean, they may have a higher risk for peri-implantitis. 

Other factors affecting peri-implantitis have been associated with selected medications predominantly involving implant prognosis including antiresorptive drugs. Dental practitioners should become increasingly aware of implant failures associated with oral bisphosphonate use. Implant failure and implant complications related to bisphosphonates are increasingly being reported [22]. With regard to the pharmacology of bisphosphates, medications that involve interruption of the homeostasis of bone can ultimately impact on implant success [23].

Periodontal disease is related to peri-implantitis. Patients with a history of periodonitis may have an increased risk of peri-implantitis [7,24]. A dysbiotic microbial community due to improper oral hygiene or other oral factors such as xerostomia may lead to quantities of red complex and orange complex bacteria around dental implants. However, patients with treated periodontitis who receive implants appear to have satisfactory implant longevity. Patients with a history of periodontitis are more likely to develop peri-implantitis [7,24].

Improper oral hygiene could result in a dysbiotic microbial flora. This dysbiotic microbial flora can include pathogenic forms of bacteria colonies [25]. Increased red complex and orange complex bacterial clusters can lead to increased risk of peri-implantitis. 

Maintenance of dental implants, especially lack of follow-up care and poor oral hygiene, are risk factors for peri-implantitis. Quarterly maintenance visits are recommended for every patient having a dental implant. Implant maintenance visits should include periodontal probings and radiographic analysis that are planned sequentially and at appropriate time intervals [26,27,28,29,30,31,32,33].

**Figure 1:**
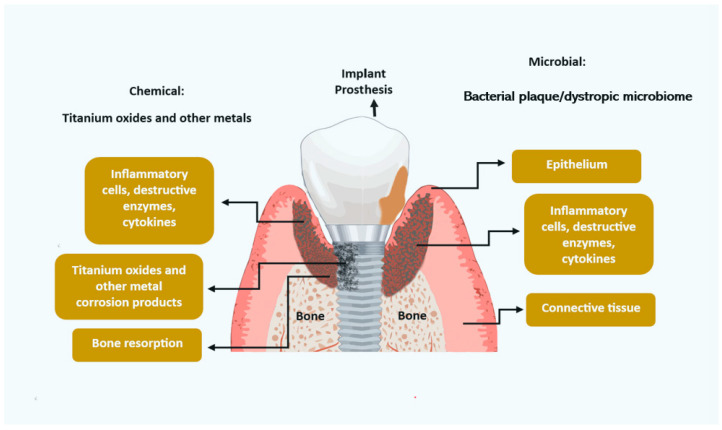
Potential etiologies for peri-implantitis.

Metal corrosion from titanium implants (Figure 1) may be an initiating factor for inflammation soft-tissue modifications and bone resorption. The mechanisms are not completely understood but may include titanium metal fatigue and stress, reaction to acidic by-products of the bacterial microbiome, chemical reactions to antimicrobial mouth rinses, mechanical damage to debridement by dental practitioners, and chemical reaction to certain diets and alcohol. Further research is required to elucidate these factors [34].

## 4. Prevalence

Peri-implantitis in the population has a reported prevalence of 1–47% and a mean prevalence of 22% [35]. The peri-implantitis prevalence affecting implants ranges from 0 to 3.4% and is most probable after 5 years of function [36]. Beyond 10 years of function, the peri-implantitis prevalence affecting implants increased to 5.8–16.9%, and the prevalence in the population increased to 10.7–47.2% [36]. The peri-implantitis prevalence is also higher in patients with a history of periodontitis and smokers [36].

The incidence of peri-implantitis affecting patients with implants was 18.8%, and the peri-implantitis incidence affecting dental implants was 9.6% [27]. Periodontal maintenance reduced peri-implantitis incidence to 14.3% [27]. The peri-implantitis incidence in smokers increased to 36.6% [27]. Compared to non-smokers, smokers have a higher peri-implantitis risk [37]. 

**Figure 2:**
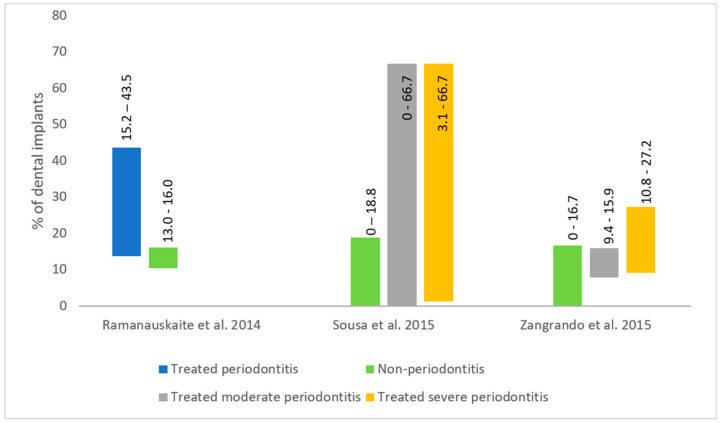
Peri-implantitis incidence based on periodontal treatment status [38,39,40].

The peri-implantitis incidence varies with periodontal health status (Figure 2). The incidence of peri-implantitis in periodontally healthy patients was reported as 10% [38]. Periodontally healthy patients have lower peri-implantitis incidence and less marginal bone loss around dental implants compared to patients with a history of periodontitis (Figure 2) [38,39,40]. In patients with aggressive periodontitis, the peri-implantitis incidence is higher at 26% [38]. Patients who have residual probings after periodontal treatment have more sites affected by peri-implantitis than treated periodontal patients without residual probings [40]. 

Patient risk factors for osteoporosis which relate to peri-implantitis may include increasing age, Asian or Caucasian ethnicity, genetic predisposition, thin or frail body type [41], poor oral hygiene and habits, smoking [42], and lifestyle lacking in exercise [43].

## 5. Pathogenesis

Bacterial challenges from dental plaque resulting in loss of attachment on dental implants proceed differently in periodontitis and peri-implantitis. This pathogenic mechanism is not clearly defined but may be related to the anatomic difference between the soft- and hard-tissue attachment around teeth and implants [44].

Several anatomical considerations may explain the difference in pathogenesis. There are two predominant reasons why implants are more susceptible to bacterial challenge than teeth, these include the attachment and vascularity around dental implants. In periodontitis around natural teeth, there is the presence of the periodontal ligament, an epithelial attachment, connective tissue attachment, and alveolar bone. In peri-implantitis around dental implants, there is the presence of epithelial attachment and alveolar bone. Epithelial attachment and bone without the periodontal ligament and the connective tissue attachment increases the dental implant susceptibility to assault by bacterial plaque [44].

In addition, the vascularity around dental implants is different from natural teeth. There are three primary sources of vascularity around teeth: alveolar bone, periodontal ligament, and periodontal soft tissues. In dental implants, there are alveolar bone and periodontal soft tissue excluding the periodontal ligament as sources of vascularity reflecting the immune response and wound healing capabilities. Since there is no periodontal ligament around dental implants, the network of vascularity including nervous bundles of sensory components is lacking. Therefore, a major source of wound healing and immune response capabilities is lacking for dental implants [44].

## 6. Immunologic Findings

Proinflammatory cytokines including TNF-α and IL-1β were statistically greater in peri-implantitis compared to peri-implant tissue in health. Increased probing depth, gingival index, and bone loss were linked to increased levels of TNF-α and IL-1β in peri-implantitis crevicular fluid. However, IL-1β levels between peri-implantitis and peri-implant mucositis were not statistically different. Other cytokines found in peri-implantitis include IL-4, IL-6,IL-8, IL-10, IL-12, and IL-17 [45].

## 7. Microbial Findings

The microbial flora in peri-implantitis is different from periodontitis [46,47]. The microflora associated with periodontitis consists of the red complex group (*Porphyromonas gingivalis*, *Tannerella forsythia*, and *Treponema denticola*), *Fusobacterium* species, *Bacteroides fragilis*, and *Prevotella intermedia* [48,49]. The microflora around implants with peri-implantitis is opportunistic and consists of Gram-negative anaerobes, asaccharolytic Gram-positive anaerobes, as well as herpesviruses like Epstein–Barr virus (EBV) [46]. 

The microflora in peri-implantitis also differs from peri-implant health. Compared to peri-implant health, the following microbial pathogens were more prevalent around implants with peri-implantitis: *Aggregatibacter actinomycetemcomitans*, *Porphyromonas gingivalis*, *Treponema denticola*, *Prevotella intermedia*, human cytomegalovirus, human herpesvirus 4 and 5, and Epstein–Barr virus 1 [50]. 

The healthy peri-implant sites have lower mean colony-forming units than peri-implantitis sites. The colony-forming units in peri-implantitis consist of opportunistic microorganisms like *Staphylococcus intermedius*, *Staphylococcus aureus*, *Streptococcus mitis*, and *Haemophilus influenzae* [51]. Furthermore, 30% of the microflora in peri-implantitis consists of *Porphyromonas gingivalis*, *Tannerella forsythia*, *Treponema socranskii*, *Staphylococcus anaerobius*, *Staphylococcus intermedius*, *Staphylococcus aureus*, and *Streptococcus mitis* [51].

## 8. Effect of Systemic Disease

The peri-implantitis risk is higher in diabetic patients [52]. Type 1 diabetes makes up 5–10% of diabetic patients, and the other 90–95% consist of Type 2 diabetes [53]. Patients with well-controlled diabetes have lower probing depths, gingival index, and bone loss compared to uncontrolled diabetes [54]. Poorly controlled diabetes increases the risk of peri-implantitis compared to well controlled diabetes or healthy patients [55].

The peri-implantitis risk is greater in cardiovascular disease patients. These patients have a higher risk of harboring Epstein–Barr virus, a herpesvirus associated with aggressive periodontal disease [56].

Based on statistical evaluation, peri-implantitis is not associated with rheumatoid arthritis [56].

Osteoporosis and osteopenia are recognized contributing factors for peri-implantitis and dental implant failures [57]. Poor bone density is a risk factor for peri-implant disease [58]. The quality of the cortical bone is related to the stability of the marginal bone around the implant for the long term [59]. Implant surgical placement in osteoporotic bone results in increased bone loss around the implant collar 1–3 years post-surgical-placement. In a large-scale clinical report on osteoporosis, periodontitis and peri-implant disease risk were reported to be elevated [57,59]. Therefore, the risk factors for osteoporosis and the medication increasing the risk of osteoporosis should be factored in for dental implant treatment planning and maintenance [43,57].

Other systemic conditions like high systolic blood pressure and obesity were also associated with peri-implantitis [60].

## 9. Impact of Medications on Peri-Implantitis

Selected medications may impact soft and hard tissue around implants [61]. Medication-related factors impacting peri-implantitis include steroids, organ transplant medications, anti-neoplastic medications, antacids, androgen-deprivation therapy (Lupron), and selective serotonin reuptake inhibitors (SSRIs).

SSRIs used to alleviate depression were reported to increase implant disease and failures [62]. The pharmacological mechanism for this observation is not clearly understood. 

Bisphosphonates were commonly used to manage osteoporosis, Paget’s disease, and breast cancer therapy, and have also been reported to be associated with implant failure. In addition, bisphosphonates and other antiresorptive medications may increase the risk of osteonecrosis in the jaw from dental implant procedures [22].

Heartburn medications like proton pump inhibitors, antacids, and H_2_ blockers can increase the risk of osteoporosis and the associated peri-implant disease [63].

## 10. Treatment of Peri-Implantitis

Peri-implantitis treatments may include adjunctive therapy and be surgical or non-surgical. Non-surgical treatment detoxifies and cleanses the implant surface with or without the utilization of adjunctive antibacterial medicaments. Non-surgical treatments used to treat peri-implantitis may include ultra-sonic or manual debridement, air-abrasion, systemic or local antimicrobial therapy, chlorhexidine therapy, local antiseptics therapy, laser therapy, and host modulation. 

Manual debridement can reduce inflammation around dental implants. Manual debridement using titanium instruments or ultrasonics showed reduced plaque and bleeding scores but had no effect on probing depths. There were also no significant treatment differences between the use of ultrasonics or titanium instruments [64]. However, repetitive treatment with oscillating brushes and curettes produced a significantly reduced bleeding index and probing depths at 6 and 12 months compared to baseline [65,66].

Air-abrasives with glycine or erythritol powder decontamination of peri-implant surfaces were shown to be comparable to ultrasonics in treating peri-implantitis [67,68,69]. Similarly, air-abrasives compared to erbium-doped yttrium aluminum garnet (Er:YAG, Biolase, Inc. Foothill, CA, USA) laser debridement reported comparable clinical results [70,71]. However, air-abrasives were more effective in reducing peri-implant inflammation when compared to chemical disinfection with chlorhexidine (CHX).

Peri-implant antimicrobial therapy with adjunctive systemic antibiotics like amoxicillin, azithromycin, or metronidazole may also have beneficial effects. Metronidazole used in conjunction with manual debridement significantly improved probing depths, clinical attachment, and bone fill compared to control 12 months after treatment [72].

Adjunctive therapy with antimicrobial therapy and antiseptic mouthrinses may improve peri-implantitis outcomes [73]. Antimicrobial therapy with debridement resulted in greater reduced probing depth compared to debridement alone [74]. Mechanical debridement with minocycline resulted in better peri-implantitis resolution compared to chlorohexidine and debridement at 12-month follow-up [75,76].

Peri-implant therapy with adjunctive antiseptic mouth rinse like chlorohexidine (CHX), sodium chloride (NaCl), hypochlorite, and herbal oral rinses may be effective against peri-implant mucositis and peri-implantitis [77,78]. However, in some peri-implantitis cases, the results may be limited [79].

Adjunctive therapy with laser disinfection may also improve peri-implantitis outcomes. Carbon dioxide (CO_2_) lasers and erbium-doped yttrium aluminum garnet (Er:YAG) lasers were found to clinically improve peri-implant parameters up to 6 months [75,80]. Er:YAG laser treatment compared to debridement with chlorhexidine resulted in greater inflammation reduction. Er:YAG lasers compared to glycine powder may produce similar clinical peri-implantitis resolution [76]. 

Laser decontamination is often used as an adjunct to manual debridement and is a useful option to decontaminate and remove bacterial biofilm on rough implant surfaces [81,82,83]. Diode lasers were reported to have some beneficial adjunctive effects [83]. Photodynamic therapy when used adjunctively for reducing peri-implant inflammation was as effective as adjunctive minocycline microspheres for up to 12 months [84].

Innovative approaches for non-surgical peri-implantitis treatment include enamel matrix (EMD) and probiotics [85,86,87]. Adjunctive use of EMD compared to manual debridement was more effective in reducing peri-implant inflammation [85]. Use of *Lactobacillus reuteri* with manual debridement produced improved clinical parameters like bleeding on probing and probing depths around implants with peri-implantitis [86]. However, oral probiotics seem to have limited effects on the microbiota around the dental implant [86].

Surface decontamination with implantoplasty or dental lasers may result in similar treatment outcomes compared to other surface decontamination techniques [75,76,88,89]. In general, non-surgical debridement with adjunctive therapy of peri-implantitis may be superior to debridement alone [74].

Non-surgical therapy was effective for debriding irritants around implants in peri-implantitis sites. This may have a positive impact on implant inflammation [65,66]. However, non-surgical therapy may not have an effect on osseous defects [90,91].

Surgical treatment involved elevation of the flap to expose the contaminated implant surface, cleaning and detoxifying with antimicrobial therapy or antiseptic solution, and grafting the osseous defects with or without bone graft and membranes. Surgical protocols may include open-flap debridement, resective osseous peri-implant procedures, or regenerative techniques. Open-flap peri-implantitis procedures may include implant debriding with ultrasonic scalers, curettes, air abrasion, curettes, burs, or laser treatment. 

Resective peri-implant procedures may include peri-implant pocket elimination and implantoplasty. After 3 yrs, the implant survival rate of implantoplasty and resective peri-implantitis treatment was reported to be 100% [92]. At 24 months, implantoplasty of contaminated implants during resective peri-implantitis treatment improved probing depth, attachment levels, and the bleeding index. However, the treatment resulted in a higher recession index in the treated implants [92].

Regenerative peri-implant techniques may include biomaterials like synthetic membranes, porcine/bovine membranes, bone graft, bone substitutes, platelet concentrates, calcium carbonate, or hydroxyapatite. Surgical peri-implantitis therapy can reduce peri-implant probing depth by 30–50% [89,93]. There are conflicting reports for the use of biomaterials in surgical peri-implantitis treatment. The use of enamel matrix derivative in the treatment of peri-implantitis showed no improvement in probing depths or bone fill [94]. Similarly, open-flap surgery with cancellous bone and 10% purified porcine collagen did not improve bleeding on probing or probing depths but significantly reduced buccal gingival recession [95].

However, other studies show that regenerative materials when used in combination with open-flap surgery were more effective in peri-implantitis resolution than without [95,96,97,98].

Regenerative procedures may produce radiographic bone fill of 2–2.17 mm [89,93,99,100]. Radiographic bone fill is often enhanced with the use of regenerative materials, but it is important to discern if the radiographic improvement equates to the clinical improvement of the peri-implant defect [101].

Laser peri-implantitis treatment may stimulate bone fill in peri-implantitis bony defects and may reduce inflammation and probing depths. The diode, Er:YAG, neodymium-doped yttrium aluminum garnet (Nd:YAG), and O_2_ lasers have studies reporting marginal bone fill in peri-implantitis defects [102].

The Nd:YAG lasers utilizing laser-assisted peri-implantitis surgical protocols may potentially rescue failing dental implants by clinical attachment gain and radiographic bone fill [103]. Nd:YAG-induced histologic evidence of regeneration in periodontitis-affected teeth may parallel the clinical response of Nd:YAG-induced peri-implantitis resolution [104,105]. Peri-implantitis treatment may be successful if treated implants report resolution of inflammation and no progressing bone loss.

With Er:YAG laser-assisted regenerative surgical therapy, although there were no significant radiographic bone changes, the probing depth was significantly improved [106].

However, there are conflicting outcomes for different peri-implantitis treatments. Heitz-Mayfield et al. reported peri-implantitis resolution in 75–93% of implants and 76–100% of patients after 12 months [107]. Their peri-implantitis treatment included non-surgical and surgical intervention with different combinations of adjunctive treatments. Other studies report less than 50% of infected implant respond to conventional surgical peri-implantitis debridement [108,109]. And guided bone regeneration with bone graft and membranes reported unpredictable outcomes [89,99,110,111].

In general, clinical attachment levels and probing depth were improved with surgical interventions compared to non-surgical approaches [7,112]. In addition, poor oral hygiene practices may have a direct negative effect on implant success [113].

## 11. Conclusions

Patients with periodontal disease or treated periodontal disease may have a higher peri-implantitis incidence. Implants in function after 5 years also have a higher peri-implantitis incidence. Smokers, patients with cardiovascular disease, and those with uncontrolled diabetes may have a higher peri-implantitis risk. Proinflammatory cytokines including TNF-α and IL-1β were significantly elevated in peri-implantitis. The microflora involved in peri-implantitis differs from periodontitis and may consist of opportunistic pathogens including *Porphyromonas gingivalis*, *Prevotella intermedia*, *Aggregatibacter actinomycetemcomitans*, *Treponema denticola*, *Treponema socranskii*, *Tannerella forsythia*, *Streptococcus mitis*, *Staphylococcus anaerobius*, *Staphylococcus aureus*, *Staphylococcus intermedius*, Epstein–Barr virus, human cytomegalovirus, and human herpesvirus 4 and 5.

All combinations of adjunctive treatments for non-surgical and surgical approaches may result in successful peri-implantitis resolution and are better than debridement alone. Surgical intervention may reduce peri-implant probing depths. However, guided bone regeneration (GBR) may be technique-sensitive and unpredictable. Meticulous post-implant maintenance may reduce peri-implantitis in high-risk patients. To determine better treatments for peri-implantitis, additional randomized controlled trials are required.
